# Diverse Lenabasum pathway activation in dermatomyositis patients’ blood

**DOI:** 10.1038/s41598-025-92001-z

**Published:** 2025-05-18

**Authors:** Nilesh Kodali, DeAnna Diaz, Rohan Dhiman, Thomas Vazquez, Rui Feng, Jay Patel, Joshua Dan, Grant Sprow, Julianne Kleitsch, Meena Sharma, Muhammad Bashir, Victoria P. Werth

**Affiliations:** 1 Department of Dermatology, University of Pennsylvania Perelman School of Medicine, Philadelphia, PA USA; 2 Corporal Michael J. Crescenz VA Medical Center, Philadelphia, PA USA

**Keywords:** Dermatomyositis, Lenabasum, IFNβ, Flow cytometry, Mechanism, Idiopathic inflammatory myopathies, Chronic inflammation

## Abstract

Lenabasum, a non-psychoactive cannabinoid type-2 receptor (CB2R) agonist, has shown promise in reducing cutaneous disease in Dermatomyositis (DM) patients. Lenabasum activates two distinct receptors: CB2R and the nuclear peroxisome proliferator-activated receptor-γ (PPARγ). Our goal was to investigate the dominant mechanism of action leading to pathogenic IFNβ reduction by lenabasum (through CB2R or PPARγ) across leukocytes. We utilized whole blood leukocytes from 14 DM patients and grouped patients as in vitro responders or non-responders. We stimulated leukocytes in vitro in the presence of CB2R and PPARγ inhibitors and lenabasum. Intracellular and extracellular marker expression was analyzed by flow cytometry. CD4^+^ T (*p* < *0.05*), monocyte-derived dendritic cells (*p* = *0.06*), and intermediate monocytes (iMs) (*p* < *0.05*) activate a CB2R-mediated lenabasum pathway in responders. Responder B cells (*p* < *0.01*), CD8^+^ T cells (*p* < *0.01*), and non-classical monocytes (*p* = *0.06*) activate a co-dependent CB2R/PPARγ-mediated lenabasum pathway. Lenabasum can independently activate CB2R or PPARγ in myeloid dendritic cells (*p* < *0.05*). Responder plasmacytoid dendritic cells (*p* < *0.05*) and classical monocytes (*p* < *0.01*) activate a PPARγ-mediated lenabasum pathway. CB2R was increased in certain responder CB2R-mediated cell populations compared to non-responders. Lenabasum elevated cyclooxygenase-2 or 15-lipoxygenase-1 levels in all responder CB2R-mediated cell populations except iMs. Baseline cell-to-cell CB2R/PPARγ testing could be useful to select ideal lenabasum candidates.

**Trial Registration:** Registered at ClinicalTrials.gov (Identifier: NCT03813160) on 2019–01-23. Sponsored by Corbus Pharmaceuticals Inc.

## Introduction

Dermatomyositis (DM) is an autoimmune connective tissue disease that mainly affects the muscle, skin, and lungs. Patients typically present with (classic DM) or without (amyopathic DM) proximal muscle involvement and are characterized by their skin manifestations such as heliotrope rashes, Gottron’s papules, and erythema in the V-neck area^1^. Research has shown that interferon-β (IFNβ) directly correlates with the Cutaneous Dermatomyositis Disease Area and Severity Index (CDASI) and has been thought to be the major driver of DM pathogenesis^2,3^. Many potential pathways culminate in IFNß and IFNγ production, but pathway markers specifically related to the type I IFN system, such as pSTING, pIRF3, pTBK1, IFNβ, and IFNγ, and pNFkB are increased in DM skin compared to HC skin^4^. The source of stimulation may vary with each patient. For instance, published work indicates that UVB irradiation of keratinocytes induces cytosolic leakage of DNA, leading to activation of the cGAS-STING pathway^5^. Work from our lab has shown that toll-like receptor 4 (TLR4) inhibition suppresses Spirulina-induced IFNγ and TNFα production in DM peripheral blood mononuclear cells (PBMCs)^5^. DM in any one patient may be the result of any one or a combination of these pathways.

Lenabasum, a non-psychoactive cannabinoid type 2 receptor (CB2R) agonist, is currently being investigated as a treatment for DM. In a phase 2 trial investigating the safety and efficacy of lenabasum on 22 DM patients, lenabasum treatment led to a significant decrease in skin activity, as measured by the CDASI that were refractory to traditional DM treatment with hydroxychloroquine^6^. Furthermore, both IFNβ and interferon-γ (IFNγ), were significantly reduced by day 85^6^. A phase 2 placebo-controlled study in skin-predominant DM patients demonstrated that the use of an IFNβ-specific antibody significantly decreased patients’ CDASI-A level at week 12 from baseline with mild adverse effects^7^. This antibody normalized gene expression in the skin of patients with DM^8^. Therefore, IFNβ and IFNγ can be used as potential markers of an anti-inflammatory cellular response to lenabasum.

**Fig. 1:**
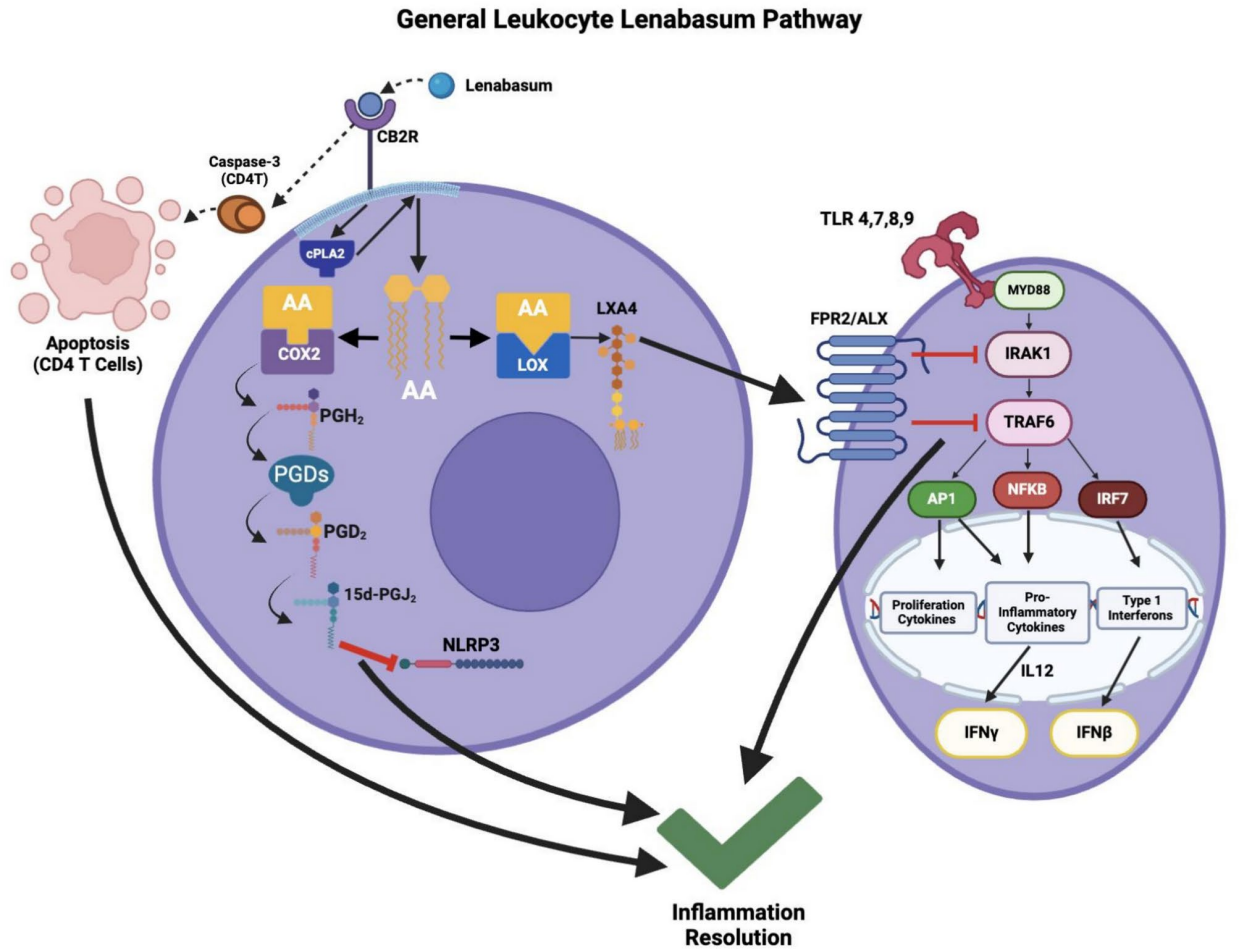
Molecular CB2R-mediated lenabasum pathway adapted from Burnstein^13^. In CD4^+^ T cell specifically, lenabasum binds to CB2R and activates caspase-3. This leads to CD4^+^ T cell apoptosis. As a general leukocyte pathway, lenabasum binds to CB2R and activates cPLA2 to break down phospholipids to AA. The pathway branches into a COX2-mediated and 15LOX1-mediated pathway. The COX2 pathway begins with the conversion of AA via COX2 into PGH_2_, which is subsequently converted to PGD_2_ by PGDs. PGD_2_ is converted to 15d-PGJ_2_ through a non-enzymatic spontaneous reaction. 15d-PGJ_2_ inhibits NLRP3 and leads to inflammation resolution. The latter 15LOX1 pathway converts AA into LXA_4_ via 15LOX1, which can bind to the FPR2/ALX receptor on a nearby cell and lead to inflammation resolution (although this LXA_4_-FPR2/ALX interaction is not definitive). FPR2/ALX exerts its anti-inflammatory effects by inhibiting IRAK1 and TRAF6. IRAK1 and TRAF6 are downstream effectors of TLR activation and ultimately lead to the production of INFβ via IRF5 and IFNγ via NFkB and IL-12^39–42^. Created with BioRender.com. cPLA2: Cytosolic phospholipase A2; AA: Arachidonic acid; COX2: Cyclooxygenase-2; 15LOX1: 15-lipoxygenase-1; PGH_2_: Prostaglandin H2; PGDs: Prostaglandin D2 synthase; PGD_2_: Prostaglandin D2; 15d-PGJ_2_: 15-Deoxy-Δ -12,14-prostaglandin J2; NLRP3: Nod-like receptor protein 3; LXA_4_: Lipoxin A4; FPR2/ALX: Formyl peptide receptor 2.

Lenabasum is also capable of binding to the nuclear receptor peroxisome proliferator-activated receptor-γ (PPARγ)^9^. Both CB2R and PPARγ have been increasingly recognized for their anti-inflammatory effects and immunomodulatory capabilities^10–16^. Recently, it has been discussed how the activation of CB2R by lenabasum increases cyclooxygenase-2 (COX2) and 15-lipoxygenase-1 (15LOX1) enzyme levels that modify arachidonic acid (AA) to produce 15-Deoxy-Δ12,14-prostaglandin J2 (15d-PGJ_2_) and lipoxin A4 (LXA_4_), which modulate nod-like receptor protein 3 (NLRP3) and human formyl peptide receptor 2 (FPR2/ALX) for their anti-inflammatory effects, respectively (Fig. 1)^17^. The full CB2R-mediated lenabasum pathway can be seen in Fig. 1. The molecular mechanism of the action of lenabasum is not completely known, and additional work is needed to confirm this putative pathway. Activation of PPARγ by lenabasum recruits co-inhibitory molecules, inhibits interleukin-8 (IL-8) promoter activity, and allow fibroblasts to differentiate into adipocytes^9^. Although lenabasum may be effective for some patients, DM patients have heterogeneous clinical presentations and display a varying response to lenabasum. Therefore, we sought to understand the different pathways lenabasum activates across different cell types to get a better understanding into the varying efficacy of lenabasum in inhibiting type 1 interferons and guide precision medicine.

## Materials and methods

### Participants

Ethical approval and signed consent were obtained from all patients prior to participating in the study through the Institutional Review Board (IRB) at the University of Pennsylvania (IRB protocol number: 808230). Our study adhered to the Declaration of Helsinki and IRB guidelines. 14 DM patients were recruited through the Department of Dermatology at the University of Pennsylvania. DM patients were diagnosed by VPW using the Sontheimer or EULAR/American College of Rheumatology (ACR) criteria. Individual participant’s demographics, treatments, and disease characteristics are listed in Table S1.

### Whole blood preparation

Whole blood was collected from DM patients in six 5-mL heparinized tubes. Immediately after collection, the tubes were inverted 8–10 times to thoroughly mix the blood with the anticoagulant. To initiate processing, the tubes were centrifuged at 500 xg for 10 min at room temperature to separate plasma from cellular components. The plasma layer was aspirated using a sterile pipette, taking care not to disturb the buffy coat or the underlying red blood cell (RBC) layer. The remaining blood, now composed of the buffy coat and RBCs, was subjected to red blood cell lysis to isolate white blood cells (WBCs).

For RBC lysis, a 1:10 dilution (whole blood volume: ACK lysing buffer volume) of ACK lysing buffer (Thermo Fisher Scientific, catalog #A10492) was added to each tube. The lysing buffer was added slowly along the inner walls of the tubes to avoid turbulence and mechanical stress to the cells. Each tube was then inverted gently 10–12 times to ensure uniform mixing of the lysing buffer with the blood. The samples were incubated at room temperature for 7 min, during which RBC lysis was confirmed by the change in color to a translucent reddish solution, indicating complete hemolysis. After incubation, the tubes were centrifuged at 300 xg for 5 min to pellet the WBCs while the lysed RBC debris remained in the supernatant. The supernatant was carefully aspirated and discarded without disturbing the WBC pellet.

The WBC pellet was resuspended in 2 mL of sterile phosphate-buffered saline (PBS, pH 7.4). The suspension was centrifuged again at 300 xg for 5 min to pellet the cells. This washing process was repeated twice more, each time resuspending the pellet in cold PBS to ensure the complete removal of residual lysing buffer and lysed RBC debris. After the final wash, the WBC pellet was resuspended in 1 mL of PBS to create a uniform cell suspension^18^.

To count and assess the viability of the isolated leukocytes, a 1:5 dilution of the cell suspension was prepared by mixing 20 µL of the WBC suspension with 80 µL of 0.4% Trypan Blue solution (Sigma-Aldrich). The mixture was pipetted to ensure uniform staining and loaded onto a hemocytometer. The cells were visualized under a light microscope at 100 × magnification, and both viable (unstained) and non-viable (blue-stained) cells were counted across four quadrants of the hemocytometer. The total leukocyte concentration was calculated using the standard hemocytometer formula, and cell viability was determined as a percentage of viable cells.

### Cell culture

Cells were immersed in cell culture medium (RPMI 1640 supplemented with 10% fetal bovine serum, 1% L-glutamine, 1% Penicillin–Streptomycin) at a density of 1 million cells per 1 mL. Cells were plated in a 24-well tissue culture plates to maintain consistent conditions across experimental wells.

To accommodate the differing stimulation requirements for dendritic and non-dendritic cell populations, two wells were prepared for each preincubation combination of CB2R/PPARγ inhibition and lenabasum treatment. This ensured that non-dendritic cells (lymphocytes and monocytes) could be stimulated with phorbol 12-myristate 13 acetate (PMA), while dendritic cell populations, which respond robustly to Toll-like receptor stimulation, could be stimulated with Resiquimod (R848).

Prior to stimulation, cells were preincubated, in various combinations, with a CB2R inhibitor [1 μM SR144528 (R&D, catolog #5039)], and a PPARγ inhibitor [10 μM T0070907 (SelleckChem, catolog #S2871)], for 1 h at 37 °C in a 5% CO_2_ humidified incubator. 15 μM of lenabasum was then added to appropriate wells for an additional hour and further incubated. Receptor inhibitors (CB2R or PPARγ) and lenabasum treatments were applied in all possible combinations to evaluate their interactions.

After preincubation with combinations of CB2R/PPARγ inhibition and lenabasum treatment, the non-dendritic cell populations were stimulated with 0.025 μg/mL of PMA, 1 μg/mL of ionomycin, and 10 μg/ml of Brefeldin A for 4 h for all wells (regardless of CB2R/PPARγ inhibition combination). For dendritic cell populations, cytokine stimulation was accomplished by adding 1 μg/mL of R848 and 10 μg/ml of Brefeldin A for 4 h for all wells (regardless of CB2R/PPARγ inhibition combination). Stimulation conditions were consistent across all wells, regardless of inhibitor or lenabasum treatment. After stimulation, culture media was removed, and cells were washed twice with 1 mL of PBS to remove excess reagents and debris prior to flow cytometry staining.

### In vitro responder vs non-responder definition

We defined our in vitro responders as patients who had at least a 50% decrease in their CD45^+^IFNβ^+^ levels after adding lenabasum. Non-responders were defined as patients who did not have a 50% decrease or had an increase in their CD45^+^IFNβ^+^ levels after adding lenabasum. These definitions were modeled after previous in vivo immunohistochemistry results from our lab that showed a 50% decrease in IFNβ after 12-week lenabasum treatment^14^.

### Flow cytometry

Plasma-removed and stimulated whole blood leukocytes were stained for flow cytometry. All cells were washed with 1 mL staining buffer (2% FBS in PBS) and immediately Fc receptor-blocked on ice for 10 min using 1 μL of pre-adsorbed H&L goat anti-mouse IgG antibody (Abcam). To minimize nonspecific binding and enhance staining consistency, 50 μL of BD Horizon Brilliant Stain Buffer was added afterwards to all tubes.

Extracellular markers were stained using the following antibodies: PerCP-Cy5.5 HLA-DR, PE-Cy7 CD11c, BV711 CD123, BUV563 CD45, BV605 CD3, BUV496 CD4, BV786 CD8, BUV 395 CD14, PE-Cy5 CD16, AF405 CB2R, Red-718 CD56, and BUV737 CD19. Staining was performed on ice and cells were subsequently incubated on ice in the dark for 30 min.

After extracellular staining, cells were washed with 1 mL of PBS and immediately fixed using 250 μL of fixation buffer per tube (BioLegend). Cells were rinsed twice with 500 μL of 1 × diluted intracellular permeabilization buffer (BioLegend) and the tubes were then stained and incubated in the dark for 20 min with the corresponding intracellular cytokine and enzyme markers: FITC IFNβ, BV650 IFNγ, AF594 ALOX15, PE 5-LOX, and AF750 COX2. Cells were subsequently washed with 1 mL of PBS and acquired within 2 days on the BD FACSymphony A3 Cell Analyzer to ensure optimal fluorescence signal. Compensation was performed using single-stained positive controls (positive and negative compensation beads for each fluorochrome), which were prepared by staining 30 min prior to data acquisition in accordance with the manufacturer’s protocol (Invitrogen AbC Total Antibody Compensation Bead Kit, Fischer Scientific). Analysis and gating were done using FlowJo v10. Wells stimulated with PMA were only gated on non-dendritic cell populations while wells stimulated with R848 were only gated on dendritic cell populations (CD11c + and CD123 +).

### Statistics

All statistics were calculated on GraphPad Prism 9.3.0. Given the non-normal distribution of the data, median values for IFNβ and IFNγ, along with their respective interquartile ranges (IQR), were utilized as the most appropriate measures of central tendency and variability. IFNβ and IFNγ comparisons across groups were made by a one-sample Wilcoxon signed-rank test. CB2R and COX2/15LOX1 levels were compared with a Mann–Whitney U test. P-values < 0.05 were considered statistically significant.

## Results

Of the 14 participants, 5 (36%) were classified as in vitro responders based on achieving a ≥ 50% reduction in CD45 + IFNβ + levels following lenabasum treatment. Among responders, 3 (60%) had amyopathic dermatomyositis, while 2 (40%) had classic dermatomyositis. In contrast, non-responders (n = 9) included 2 (22%) with amyopathic dermatomyositis and 7 (78%) with classic dermatomyositis (Table S1).

Responders were predominantly antibody-negative, with 4 out of 5 (80%) lacking detectable autoantibodies, compared to non-responders, where 5 out of 9 (56%) were antibody-positive (including SAE1, MDA5, and P155/140). Median CDASI-A scores were slightly lower in responders (15; IQR 12–18) compared to non-responders (20; IQR 11–33), although not statistically significant (p = 0.0932). Disease duration did not show significant differences, with responders having a median duration of 83 months (IQR 68–176) and non-responders 99 months (IQR 26–210). Interstitial lung disease (ILD) was identified in 1 responder (20%) and 2 non-responders (22%) (Table S1).

Overall, no statistically significant differences in baseline clinical characteristics, including DM subtype, CDASI-A scores, or disease duration, were observed between in vitro responders and non-responders.

### Increased responder CB2R levels in certain cell populations

**Fig. 2:**
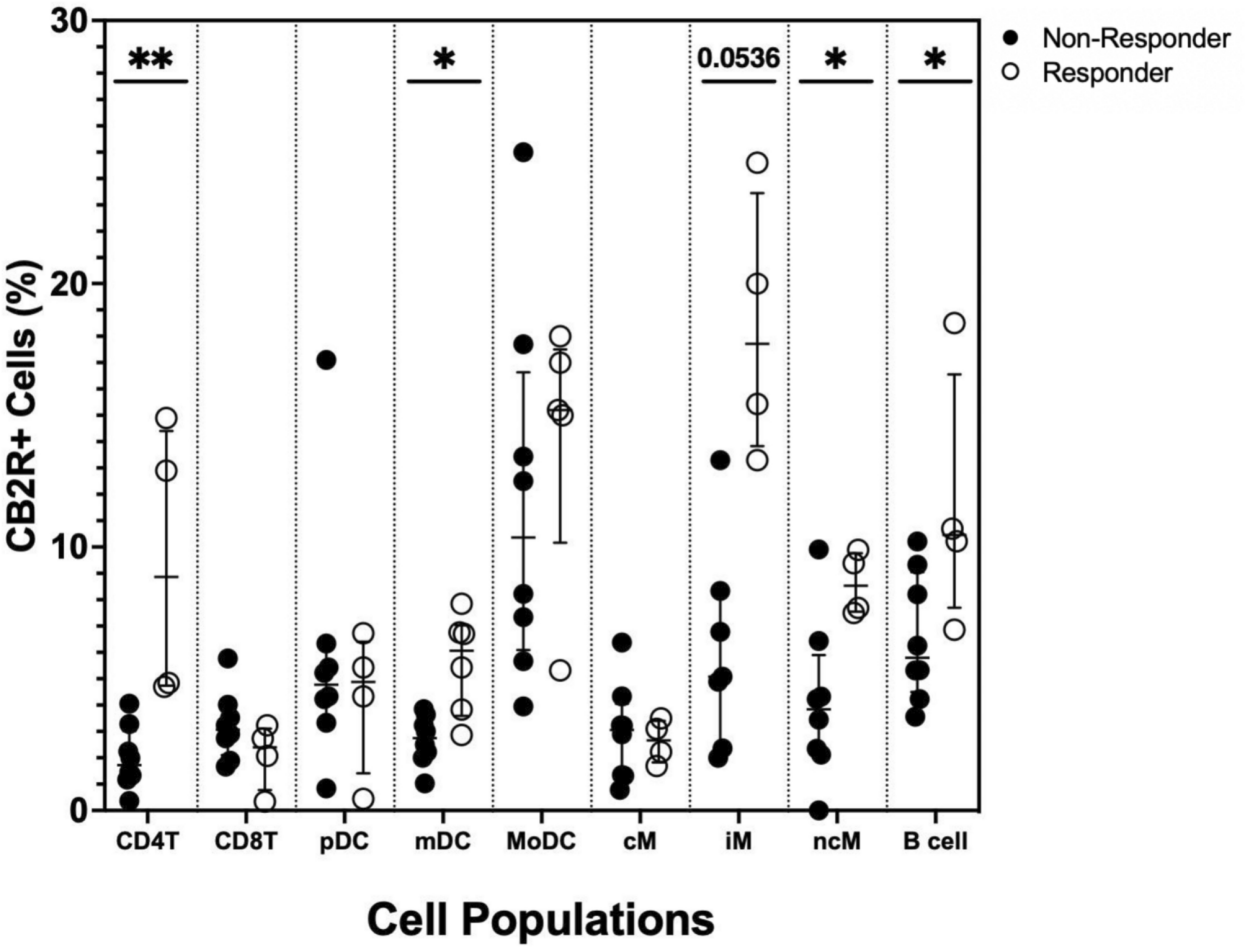
Flow cytometry of DM whole blood leukocytes displaying percent positivity of CB2R across various cell populations in non-responders and responders after stimulation with 0.025 μg/mL of PMA, 1 μg/mL of ionomycin, and 10 μg/ml of Brefeldin A for 4 h for non-dendritic cell populations and 1 μg/mL of R848 and 10 μg/ml of Brefeldin A for 4 h for dendritic cell populations. Graph line resembles the median. Lines above and below the median represent the interquartile ranges. CD4^+^ T cells (*p* < *0.01*), mDCs (*p* < *0.05*), iMs (*p* = *0.0536*), ncMs (*p* < *0.01*), and B cells (*p* < *0.01*) have increased CB2R levels in responders.

Whole blood leukocytes were gated for CB2R levels using the percent of parent positive for CB2R on individual cell populations. Full flow cytometry parent and cytokine gating can be seen in Figs. S1 and S2. CD4^+^ T cells (Median, IQR) [8.87% (4.81–13.4) vs 1.73% (1.29–2.5); *p* < *0.01*], myeloid dendritic cells (mDCs) [6.06% (4.23–6.75) vs 2.75% (2.17–3.33); *p* < *0.05*], non-classical monocytes (ncMs) [8.54% (7.64–9.51) vs 3.84% (2.29–4.85); *p* < *0.05*], and B cells [10.46% (9.38–12.65) vs 5.80% (5.05–8.49); *p* < *0.05*] all had significantly higher CB2R levels in the responders when compared to the non-responders (Fig. 2). Intermediate monocytes (iMs) followed this pattern with a trending increase in CB2R levels [17.7% (14.9–21.15) vs 5.08% (3.63–7.56); *p* = *0.05*] in responders compared to non-responders (Fig. 2).

### ***CD4***^+^***T cells, moDCs, and iMs demonstrate a CB2R-mediated lenabasum pathway***

**Fig. 3:**
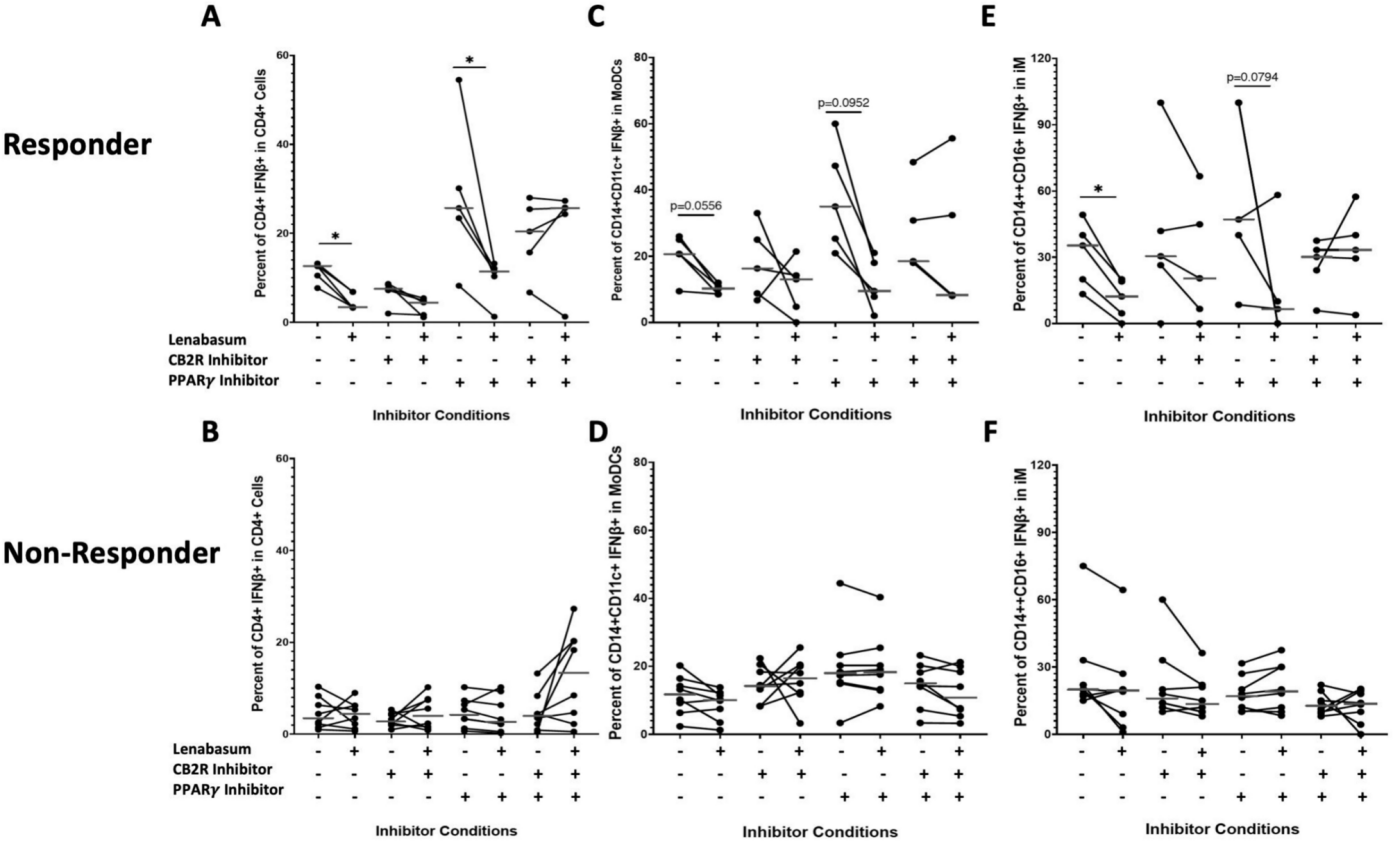
Flow cytometry of DM whole blood leukocytes displaying percent positivity of IFNβ across CD4^+^ T, moDCs, and iMs in non-responders and responders with various CB2R/PPARγ inhibitors and lenabasum treatment. Black line reflects the median. CD4^+^ T, moDCs, and iMs display a CB2R-mediated lenabasum pathway. Non-responders were used as controls and showed no difference in IFNβ positivity in CD4^+^ T, moDCs, and iMs with lenabasum treatment and CB2R/PPARγ inhibition. (**A**) IFNβ positivity in CD4T^+^ cells in responders after lenabasum treatment and CB2R/PPARγ inhibition. (**B**) IFNβ positivity in moDCs in responders with lenabasum treatment and CB2R/PPARγ inhibition. (**C**) IFNβ positivity in iMs in responders with lenabasum treatment and CB2R/PPARγ inhibition. (**D**) IFNβ positivity in CD4^+^ T in non-responders with lenabasum treatment and CB2R/PPARγ inhibition. (**E**) IFNβ positivity in moDCs in non-responders with lenabasum treatment and CB2R/PPARγ inhibition. (**F**) IFNβ positivity in iMs in non-responders with lenabasum treatment and CB2R/PPARγ inhibition.

Using various pretreatment combinations of CB2R and PPARγ inhibitors with and without lenabasum in responders, individual cell populations were double gated on IFNβ to determine which receptor lenabasum activates to decrease IFNβ production. CD4^+^ T cells in responders showed a significant decrease in IFNβ positivity when treated with lenabasum (Median, IQR) [12.6% (10.5–12.67) to 3.46% (3.3–6.8); *p* < *0.05*], while CD4^+^ T cells did not significantly decrease IFNβ production [7.56% (7.21–8.45) to 4.46% (1.66–4.89); *p* > *0.05*] upon lenabasum treatment when only CB2R was inhibited in responders (Fig. 3a). When PPARγ was inhibited, CD4^+^ T cells in responders still showed a significant decrease in IFNβ when treated with lenabasum [25.7% (23.43–30.12) to 11.43% (10.32–12.1); *p* < *0.05*] (Fig. 3a). The same diminished anti-inflammatory response was seen when both CB2R and PPARγ were inhibited, where there was a non-significant increase in IFNβ in CD4^+^ T cells when treated with lenabasum [20.43% (15.7–25.43) to 25.67% (24.32–25.8); *p* > *0.05*] (Fig. 3a). In non-responders, there was no significant difference in IFNβ production in CD4^+^ T cells across all the various CB2R/PPARγ inhibitions with and without lenabasum (Fig. 3b).

Similar to the CD4^+^ T cells, monocyte-derived dendritic cells (moDCs) did not have a decrease in IFNβ levels in responders when inhibiting CB2R. With no receptor inhibitions, moDCs showed a trend towards a significant decrease in IFNβ production when treated with lenabasum [20.7% (20.6–25) to 10.2% (8.57–10.9); *p* = *0.06*] (Fig. 3c). When only CB2R was inhibited, there was no significant difference in moDC IFNβ production when lenabasum [16.3% (8.77–25) to 13% (4.73–14.2); *p* > *0.05*] was added (Fig. 3c). With PPARγ inhibition, moDCs continued to show a trending decrease in IFNβ production when adding lenabasum [35% (25.3–47.3) to 9.48% (7.76–18); *p* = *0.10*] (Fig. 3c). Similarly, when CB2R and PPARγ were inhibited together, there was a non-significant decrease in moDC IFNβ production when adding lenabasum [18.5% (18.3–30.8) to 8.29% (8.2–32.4); *p* > *0.05*] (Fig. 3c). In non-responders, IFNβ levels in moDCs were unaffected by the combination of inhibitors and lenabasum treatment (Fig. 3d).

Intermediate monocytes followed the same trend as CD4^+^ T cells and moDCs, where the anti-inflammatory response in responders was diminished when inhibiting CB2R. With no inhibitions in responders, there was a significant decrease in iM IFNβ production when adding lenabasum [35.32% (20–40) to 12.23% (4.56–19); *p* < *0.05*] (Fig. 3e). When only CB2R was inhibited, there was a nonsignificant difference in iM IFNβ production when lenabasum was added [30.45% (26.4–41.9) to 20.45% (6.56–44.9); *p* > *0.05*] (Fig. 3e). When PPARγ was inhibited and lenabasum was added, IFNβ production in iMs had a trending decrease [47.1% (40–100) to 6.54% (0–10); *p* = *0.08*] (Fig. 3e). When CB2R and PPARγ were inhibited together, there was also a nonsignificant difference in iM IFNβ production with lenabasum treatment [30.12% (24.1–33.33) to 33.3% (29.45–40); *p* > *0.05*] (Fig. 3e). Non-responders showed no difference in iM IFNβ levels across all inhibitor and lenabasum treatments (Fig. 3f).

### ***B cells, CD8***^+^***T cells, and ncMs demonstrate a CB2R and PPARγ mediated lenabasum pathway***

**Fig. 4:**
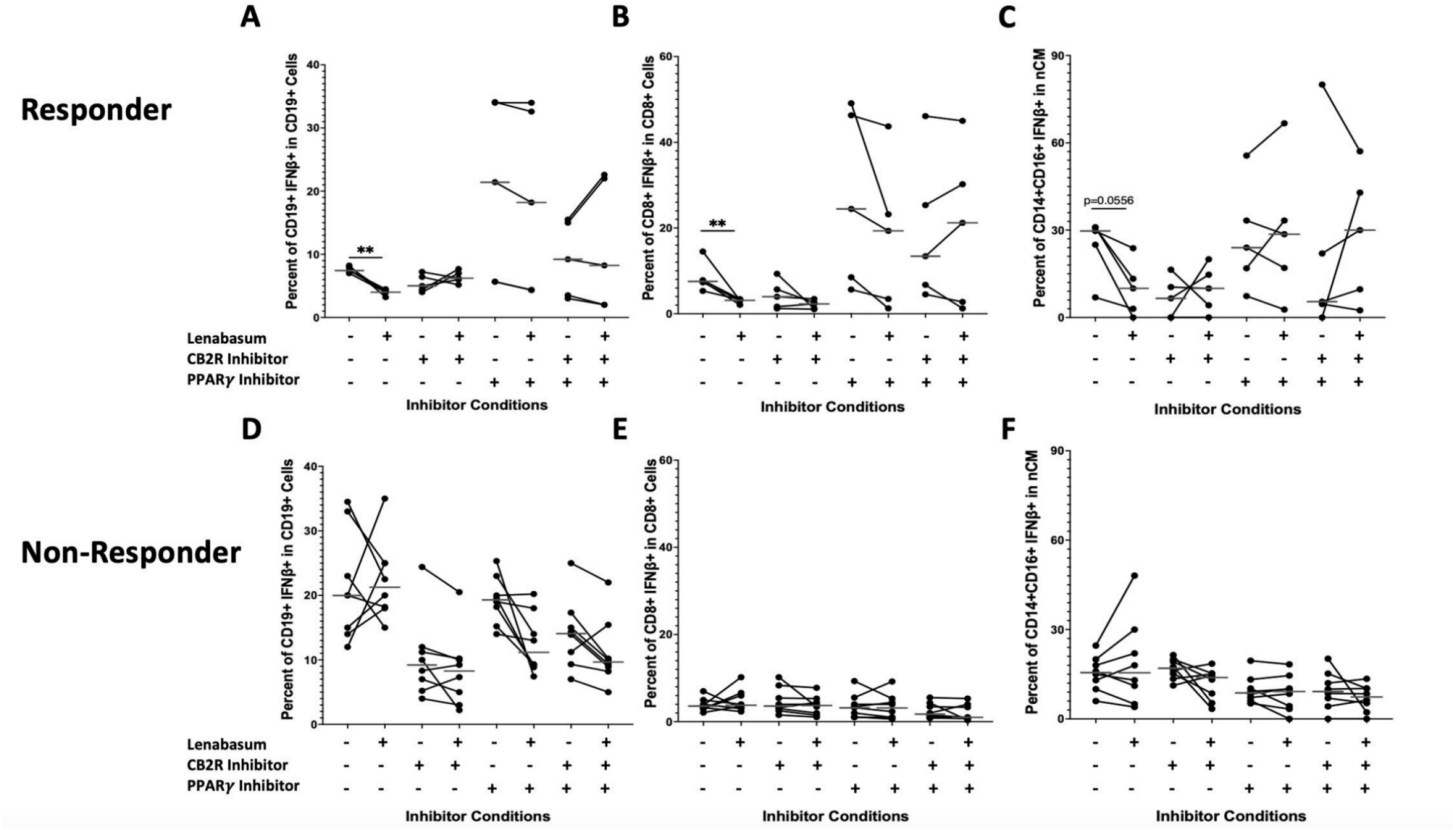
Flow cytometry of DM whole blood leukocytes displaying percent positivity of IFNβ across B cells, CD8^+^ T, and ncMs in non-responders and responders with various CB2R/PPARγ inhibition and lenabasum treatment. Black line reflects the median. B cells, CD8^+^ T, and ncMs display a co-dependent CB2R/PPARγ-mediated lenabasum pathway. Non-responders were used as controls and showed no difference in IFNβ positivity in B cells, CD8^+^ T cells, and ncM with lenabasum treatment and CB2R/PPARγ inhibition. (**A**) IFNβ positivity in B cells in responders with lenabasum treatment and CB2R/PPARγ inhibition. (**B**) IFNβ positivity in CD8^+^ T in responders with lenabasum treatment and CB2R/PPARγ inhibition. (**C**) IFNβ positivity in ncMs in responders with lenabasum treatment and CB2R/PPARγ inhibition. (**D**) IFNβ positivity in B cells in non-responders with lenabasum treatment and CB2R/PPARγ inhibition. (**E**) IFNβ positivity in CD8^+^ T cells in non-responders with lenabasum treatment and CB2R/PPARγ inhibition. (**F**) IFNβ positivity in ncMs in non-responders with lenabasum treatment and CB2R/PPARγ inhibition.

In responders, B cells, CD8^+^ T cells, and ncMs displayed a co-dependence on both the CB2R-mediated and PPARγ-mediated lenabasum pathways. Without both CB2R and PPARγ inhibition and after lenabasum treatment, there was a significant decrease in IFNβ production in B cells [7.46% (7.44–8) to 4% (4–4.15); *p* < *0.01*] (Fig. 4a) and CD8^+^ T cells [7.55% (7.32–7.82) to 3.11% (3.11–3.29); *p* < *0.01*] (Fig. 4b) with a trending decrease in ncMs [29.7% (25.00–30.80) to 10% (3–13.3); *p* = *0.0556*] (Fig. 4c). When CB2R and PPARγ were inhibited either independently or together, the lenabasum treatment caused a non-significant decrease (and in some instances, a nonsignificant increase) in IFNβ levels in responder B cells (Fig. 4a), CD8^+^ T cells (Fig. 4b), and ncMs (Fig. 4c). Non-responders demonstrated no difference in IFNβ production across all inhibitors and lenabasum treatment groups in the B cells, CD8^+^ T cells, and ncMs (Fig. 4d–f).

### Myeloid dendritic cells activate a CB2R-mediated or PPARγ-mediated lenabasum pathway

**Fig. 5:**
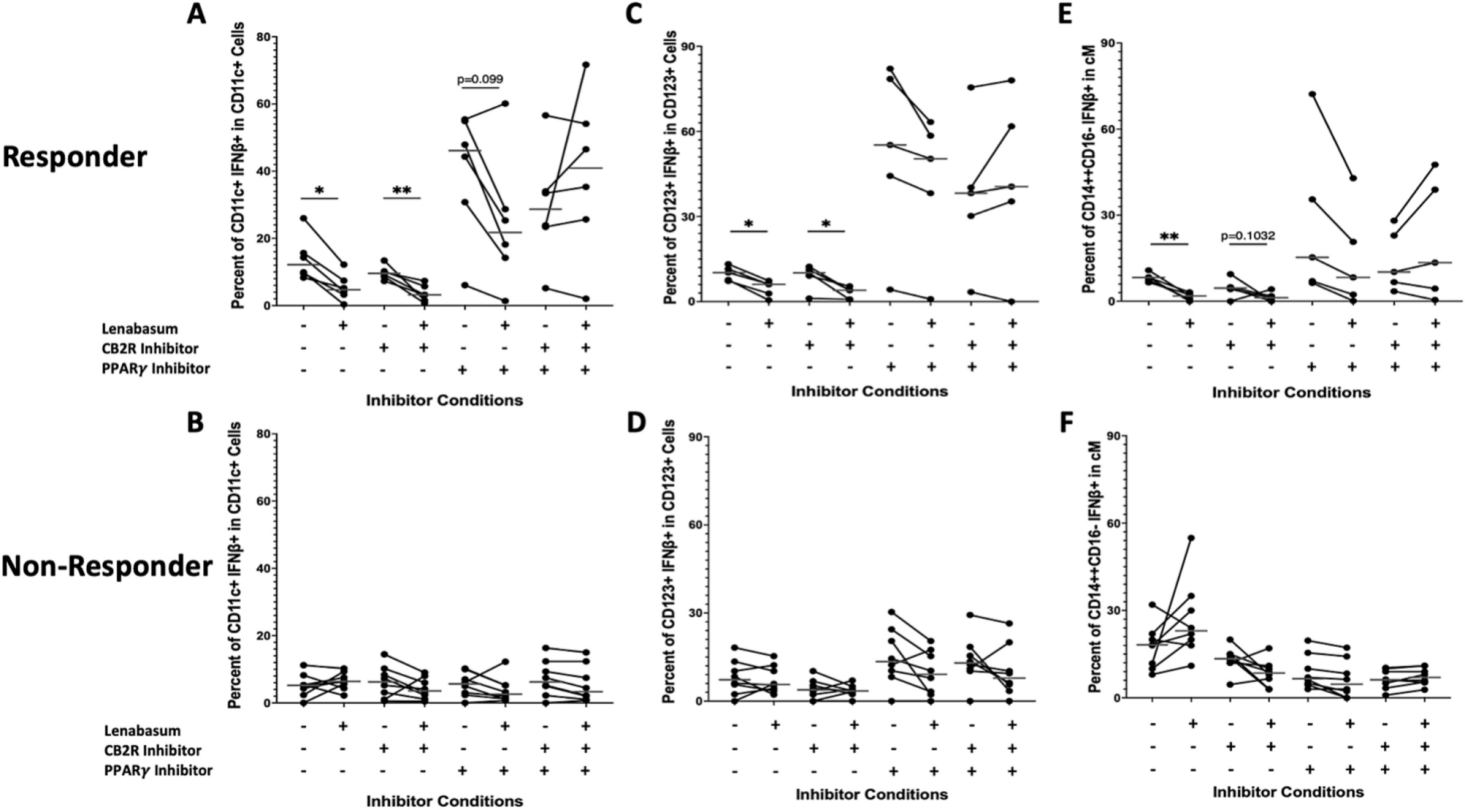
Flow cytometry of DM whole blood leukocytes displaying percent positivity of IFNβ in mDCs in non-responders and responders with various CB2R/PPARγ inhibition and lenabasum treatment. Myeloid dendritic cells display an independent CB2R or PPARγ-mediated lenabasum pathway. Plasmacytoid dendritic cells and classical monocytes display a PPARγ-mediated lenabasum pathway. Non-responders were controls and showed no difference in mDC, pDC, and cM IFNβ positivity with lenabasum treatment and CB2R/PPARγ inhibition. (**A**) IFNβ positivity in mDCs in responders with lenabasum treatment and CB2R/PPARγ inhibition. (**B**) IFNβ positivity in pDCs in responders with lenabasum treatment and CB2R/PPARγ inhibition. (**C**) IFNβ positivity in cMs in responders with lenabasum treatment and CB2R/PPARγ inhibition. (**D**) IFNβ positivity in mDCs in non-responders with lenabasum treatment and CB2R/PPARγ inhibition. (**E**) IFNβ positivity in pDCs in non-responders with lenabasum treatment and CB2R/PPARγ inhibition. (**F**) IFNβ positivity in cMs in non-responders with lenabasum treatment and CB2R/PPARγ inhibition.

Out of all the cell populations investigated in responders, mDCs were the only population that showed the capability to activate either CB2R OR PPARγ when treated with lenabasum, although the results were not statistically significant. With no CB2R or PPARγ inhibition, lenabasum treatment caused a significant decrease in mDC IFNβ production [12.13% (9.69–15.32) to 4.72% (3.51–6.88); *p* < *0.05*] (Fig. 5a). With only CB2R inhibition and lenabasum treatment, there was still a significant decrease in mDC IFNβ production [9.55% (8.76–10.13) to 3.22% (1.98–5.13); *p* < *0.01*] (Fig. 5a). Likewise, with PPARγ inhibition alone, a trending decrease in mDC IFNβ production was seen with lenabasum treatment [46.11% (34.17–53.15) to 21.78% (15.23–27.86); *p* = *0.10*] (Fig. 5a). However, when both CB2R and PPARγ were inhibited together, lenabasum treatment resulted in a nonsignificant increase in mDC IFNβ production [28.67% (23.55–33.86) to 40.91% (28.08–52.2); *p* > *0.05*] (Fig. 5a). Myeloid dendritic cells in non-responders followed the same trend as other cell populations and had no difference in IFNβ production across all inhibition and treatment groups (Fig. 5b).

### pDCs and cMs activate a PPARγ mediated lenabasum pathway

Plasmacytoid DCs (pDCs) and classical monocytes (cMs) in responders showed a diminished lenabasum response when inhibiting PPARγ. Without any inhibition, lenabasum treatment resulted in a significant reduction in both pDC [10.21% (7.65–11.5) to 6.06% (2.86–6.21); *p* < *0.05*] and cM [8.32% (7.38–8.33) to 1.85% (0.69–2.33); *p* < *0.01*] IFNβ levels (Fig. 5c,e). With CB2R inhibition alone and lenabasum treatment, pDCs had a significant decrease [10.1% (9.11–11.21) to 4% (0.81–4); *p* < *0.05*] and cMs had a trending decrease [4.62% (4.21–4.92) to 1.23% (0.14–1.79); *p* = *0.10*] in IFNβ levels (Fig. 5c,e). When PPARγ was inhibited independently or in combination with CB2R, lenabasum treatment resulted in nonsignificant decreases or slight increases in IFNβ production from pDCs and cMs (Fig. 5c,e). Much like other cell populations in non-responders, the non-responder IFNβ production in pDCs and cMs was unaffected by the CB2R/PPARγ inhibitions and lenabasum treatment (Fig. 5d,f).

### Diverse COX2/15LOX1 recruitment in CB2R-mediated lenabasum pathway activated cells

**Fig. 6:**
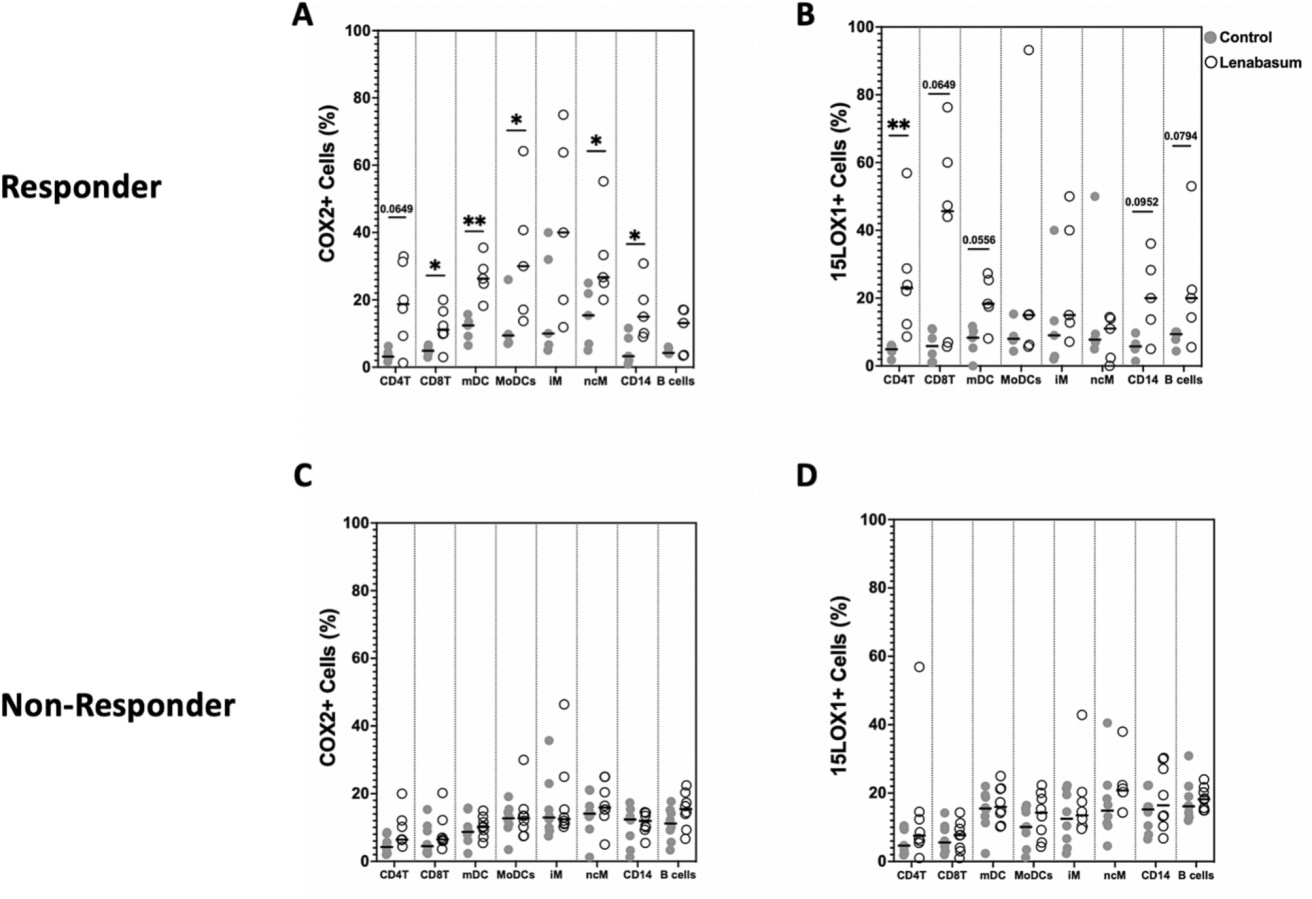
Flow cytometry of DM whole blood leukocytes displaying percent positivity of COX2 and 15LOX1 across various cell populations in non-responders and responders with lenabasum treatment. Graph line resembles the median. (**A**) COX2 positivity across various cell populations in responders with lenabasum treatment. (**B**) 15LOX1 positivity across various cell populations in responders with lenabasum treatment. (**C**) COX2 positivity across various cell populations in non-responders with lenabasum treatment. (**D**) 15LOX1 positivity across various cell populations in non-responders with lenabasum treatment.

COX2 and 15LOX1 enzymes were recruited upon lenabasum treatment and have been associated with lenabasum-specific CB2R activation. We compared the COX2 and 15LOX1 levels with and without lenabasum in the CB2R-mediated cell populations to understand the downstream cell-specific activation mechanism of lenabasum. In responders, there was a significant and trending increase in both COX2 and 15LOX1 when treated with lenabasum in CD4^+^ T cells [COX2: 3.14% (2.77–4.1) to 18.72% (11.35–28.49); *p* = *0.06*] [15LOX1: 4.92% (4.3–5.6) to 23.01% (14.77–27.55); *p* < *0.01*], CD8^+^ T cells [COX2: 4.89% (4.34–5.75) to 11.15% (9.91–15.53); *p* < *0.05*] [15LOX1: 5.85% (1.72–10.14) to 45.65% (16.21–56.83); *p* = *0.0649*], mDCs [COX2: 12.4% (9.21–13.44) to 26.32% (24.8–29.2); *p* < *0.01*] [15LOX1: 8.32% (5.32–10.21) to 18.32% (17.5–25.32); *p* = *0.06*], and CD14^+^ cells [COX2: 3.26% (2–8.63) to 15% (10.1–20); *p* < *0.05*] [15LOX1: 5.76% (5–6.49) to 20% (13.7–28.3); *p* = *0.0952*] (Fig. 6a,b). Responder moDCs [COX2: 9.43% (7.34–9.78) to 30% (17.1–40.7); *p* < *0.05*] and ncMs [COX2: 15.4% (6.9–21.9) to 26.7% (25–33.3); *p* < *0.05*] only showed significant increases in COX2, but not 15LOX1, levels when treated with lenabasum (Fig. 6a). On the other hand, B cells had a trending increase in 15LOX1 [9.39% (7.81–10) to 20% (14.3–22.5); *p* = *0.08*], but not COX2 levels, when treated with lenabasum (Fig. 6b). Intermediate monocytes were the only CB2R-mediated cell population investigated that did not have increased COX2 and 15LOX1 levels after adding lenabasum in responders (Fig. 6a,b). Non-responders showed no difference in COX2 and 15LOX1 levels upon lenabasum treatment across all cell populations (Fig. 6c,d).

When stratifying the cohort by disease duration (< 50 months vs. ≥ 50 months), drug treatments, or clinical DM subtypes (amyopathic vs classic DM), we did not observe significant differences in IFNβ responses to lenabasum within these subgroups. These specific clinical variables did not appear to influence IFNβ reductions in our study. Furthermore, cellular IFNγ responses were not significant for any cell stimulus/inhibitor across responders and non-responders (Figs. S3, S4, S5).

## Discussion

In this study, we demonstrate cell specific responses to lenabasum resulting in differential CB2R and PPARγ-mediated IFNβ reduction across leukocytes in DM. Using flow cytometry to quantify the levels of CB2R, IFNβ, COX2, and 15LOX1 across various cell populations in lenabasum responders vs non-responders, we highlight selective efficacy in responders based on contrasting single cell pathway activation. Responders demonstrated a significant CB2R or PPARγ IFNβ reduction across immune populations. Non-responders, however, showed high variance in cellular IFNβ before/after treatment with no significant changes.

CD4^+^ T, moDCs, and iMs showed a predominantly CB2R-mediated pathway activation. In the skin, we have previously shown that CD4^+^ T cells are important in the pathogenesis of DM because of their increased recruitment and extensive IFNβ and IL-31 production, both of which we have shown to be decreased in tissue after lenabasum treatment in a phase 2 clinical trial^6,14^. Our findings are supported by CB2R-mediated cytokine reduction from CD4^+^ T cells treated with lenabasum in a multiple sclerosis model^19^.

CB2R-mediated lenabasum activity was shown to be essential in inhibiting moDC chemotaxis to inflammatory sites by reducing CCR2, CCR1, and IL-1β, demonstrating the potential for both cytokine production and cell migration inhibition in DM^20^.

In various idiopathic myopathies, iMs were found to be increased in the circulation with increased TLR 2/4 and pro-inflammatory IL-6 production^21^. IMs in chronic inflammatory diseases were shown to also produce the highest amounts of antigen-presenting molecules, such as HLA-DR, HLA-DP, and HLA-DQ, among monocytes^22^. With the increasing relevance of iMs in the progression of autoimmune inflammatory conditions such as DM, it is important to understand the mechanism of action of lenabasum for potential therapeutic baseline CB2R testing to determine ideal lenabasum candidates.

Plasmacytoid DCs (pDCs) and cMs display a predominantly PPARγ-mediated lenabasum mechanism. While few pDCs are present in the skin tissue of DM patients, their systemic effects, including increased expression of IFNγ and IFNβ, highlight their distinct role in DM pathogenesis^4^. Furthermore, their presence in muscle tissue has been demonstrated, particularly in juvenile DM. PDCs were found to be abundant in inflamed muscle tissue, often in perivascular regions, and expressed markers of activation such as CD83, indicating their potential involvement in local inflammation and immune activation^23^. A PPARγ-mediated lenabasum response in pDCs has been shown in a multiple sclerosis model, where treatment with lenabasum resulted in a trending decrease in TNFα and IFNα, with or without CB2R inhibition. This suggests an anti-inflammatory response via PPARγ that is compatible with the PPARγ-dominant lenabasum effect on DM pDCs^24^.

Classical monocytes are the majority of circulating monocytes and function in phagocytosis and innate immune sensing. Due to their ability to initially respond to pathogens, initiate acute inflammation, and differentiate into pro-inflammatory cells (such as macrophages), they are commonly known to contribute to chronic inflammatory diseases^22^. In idiopathic inflammatory myopathies, there are fewer classical monocytes circulating but these cells express increased amounts of TLR4^21^. Although there has been no study to directly investigate the role of cMs in DM, there is evidence of increased CB2R on overall monocytes in DM skin^12^. Also spirulina, a trigger of DM that works through TLR4, is associated with increased TLR4 in the skin relative to other forms of DM^25^. Therefore, understanding the mechanism of lenabasum on cMs may assist future monocyte-driven studies and therapeutic endeavors.

B cells, CD8T^+^, and ncMs show a co-dependence on both the CB2R-mediated and PPARγ-mediated lenabasum mechanism. CB2R and PPARγ cross-talk and co-dependence has been shown in an inflammatory arthritis study in which BCP, a selective CB2R molecule, upregulates PPARγ in a CB2R-mediated mechanism. Blocking CB2R resulted in the decreased anti-inflammatory effects of BCP via both CB2R and PPARγ^26^. In lenabasum treatment, this co-dependence could be due to the increase of CB2R-induced COX2, 15LOX1, 15d-PJ_2_, and LXA_4_, which have shown the capability to modulate PPARγ and act as PPARγ agonists^27–30^. This warrants further investigation into the lenabasum-mediated cross-talk mechanisms between CB2R and PPARγ.

In DM, B cells are important not only for their role in autoantibody production but also for their cytokine-mediated contributions to inflammation. Although traditionally considered low in number within the inflammatory infiltrate of DM skin, recent studies from our lab demonstrate an increased presence of CD20 + and CD38 + B cells in DM skin compared to healthy controls. These B cells produce pro-inflammatory cytokines, including IL-6 and IFNγ, which play critical roles in perpetuating the inflammation in DM^31^. Furthermore, regulatory B cells that produce TGFβ are also elevated in DM, indicating a dual role for B cells in both promoting and modulating inflammation. Interestingly, PPARγ and CB2R are both highly expressed in B cells in DM skin, as previously reported^4,14^, aligning with lenabasum’s dual-targeting mechanism to modulate these pathways. This evidence positions lenabasum as a potential therapeutic agent to address the dysregulated inflammatory responses mediated by B cells.

CD8^+^ T cells in DM are increased in muscle and skin with higher production of IFNβ. It has been thought that they contribute towards autoimmunity and cytotoxicity through the release of perforin and Granzyme B^32^. Lastly, ncMs are part of the first line of defense by exerting an anti-inflammatory effect and by clearance of pathogens^33^. However, in SLE, a similar disease driven by type-1 IFNs, ncMs were shown to have inflammatory properties (through the release of cytokines) and antigen-presenting capabilities through expression of HLA-DR, CD80, and CD86^34^. Due to the pathogenic ability of these cell populations, characterizing baseline levels of both CB2R and PPARγ in B cells, CD8^+^ T cells, and ncMs could be useful in determining the efficacy of lenabasum treatment.

Lenabasum demonstrated a unique ability to activate either CB2R or PPARγ independently in mDCs. Οur lab has shown that mDCs are a major driver of DM through their increased cell count in DM skin, high production of IFNβ and IL-31 (a driver of itch in DM), and activation of IRF3 (an upstream pathway marker involved in the induction of type-1 IFNs). Myeloid DCs also interact with and modulate CD4^+^ T cells, CD8^+^ T cells, natural killer (NK), CD14^+^CD16^+^ macrophages, and FOXP3^+^ T cells^16^. In fact, mDCs have been associated with hydroxychloroquine treatment refractoriness^35^. Therefore, lenabasum’s ability to target one of the major drivers of DM (mDCs) through multiple different pathways supports its therapeutic use as an efficacious DM treatment.

CD4^+^ T, mDCs, iMs, ncMs, and B cells all showed a significantly increased baseline CB2R in responders, specifically among the cell populations that primarily activated a CB2R-mediated lenabasum pathway. This was further supported by downstream recruitment of COX2 and 15LOX1 in a CB2R-lenabasum mediated pathway. We suspect non-responders showed no variance in IFNβ in response to the CB2R inhibition because of their lower baseline levels of CB2R in CB2R-dependent cell populations. Plasmacytoid DCs and cMs, which activate a PPARγ-mediated lenabasum pathway, did not have an increase in responder CB2R levels. Baseline decreases in cellular CB2R may explain refractoriness to lenabasum treatment.

Downstream of CB2R, there seems to be heterogeneity in terms of which pathway the mechanism branched off into (COX2 or 15LOX1). CD4^+^ T, CD8^+^ T, mDCs, and CD14^+^ cells all showed recruitment and activation of both COX2 and 15LOX1. MoDCs and ncMs significantly recruited only COX2 enzymes. B cells were the only cells that displayed CB2R-mediated lenabasum recruitment of 15LOX1. Intermediate monocytes were the only cell population investigated that activated the CB2R-mediated lenabasum pathway with no recruitment of COX2 and 15LOX1. This could indicate that an alternative pathway in iMs is being activated downstream of CB2R or there is cross-talk with another downstream pathway that produces the anti-inflammatory effects. This warrants further investigation into downstream lenabasum mechanisms in iMs. Understanding these cell-specific downstream pathways will assist future therapeutic testing to determine lenabasum’s specificity and applicability to both cohorts of patients and disease processes.

These findings complement clinical observations from the Phase 3 DETERMINE trial, which assessed lenabasum’s efficacy and safety in DM. While the trial did not achieve its primary or secondary endpoints across the overall DM population, subgroup analyses revealed significant improvements in skin-related outcomes, including rash severity, particularly among amyopathic DM patients^36^. This subgroup demonstrated responses comparable to those observed in the Phase 2 trial, where lenabasum showed pronounced reductions in skin disease activity^6^.

Building on these observations, the open-label extension (OLE) of the NCT02466243 trial demonstrated that lenabasum reduced CDASI activity scores by 21.8 points at week 68, with 58.3% of patients maintaining stable disease for at least three years after discontinuing treatment—significantly higher than the 20% in controls (P = 0.035)^37^. While that study established CB2R activation as a key anti-inflammatory mechanism, it did not account for heterogeneity in patient response. Our analysis addresses this gap, revealing that responders exhibited higher baseline CB2R expression, suggesting that receptor availability may drive treatment efficacy and long-term disease stability.

This mechanistic insight provides crucial context for the clinical outcomes observed in OLE patients. Specifically, the heightened baseline CB2R expression in lenabasum responders, found in CD4 + T cells and moDCs, suggests that receptor availability is a key determinant of therapeutic efficacy. These CB2R-dominant populations demonstrated robust IFNβ reductions, reinforcing the idea that lenabasum’s efficacy is closely tied to CB2R-mediated pathways. The improved outcomes in amyopathic DM patients may reflect their disease’s greater reliance on these immune pathways, which lenabasum effectively modulates.

Furthermore, the differences in treatment responses between amyopathic and classic DM patients underscore the need for patient stratification based on immune profiles. By identifying receptor expression and pathway dependencies as potential biomarkers, future studies can refine therapeutic approaches, optimizing lenabasum’s efficacy for specific DM subgroups. These insights not only explain the clinical variability observed in the Phase 3 trial, but also emphasize the importance of mechanistic understanding to guide personalized treatment strategies in DM.

This study is not without its limitations. The cohort is relatively small with 14 DM patients, 5 of whom were responders all recruited from the same institution. The heterogeneity of antibodies that were positive and lack of substantial antibody positivity made it difficult to compare antibody profile between responders and nonresponders. The definition of responders vs nonresponders utilized in this study relies on a 50% reduction in IFNβ, an approximation modelled on prior in vivo studies, but may not fully reflect true in vivo clinical responsiveness, as other immune pathways or cytokines could contribute to patient heterogeneity in response to lenabasum. While we observed higher CB2R levels in responders compared to non-responders, the absence of healthy controls restricts our ability to fully contextualize these findings relative to normal baseline levels. Prior studies from our lab, however, have shown significantly elevated CB2R expression in DM lesional skin and PBMCs compared to healthy controls, particularly in pDCs and mDCs^14^. This suggests that CB2R upregulation in DM is an established phenomenon. However, our study does not establish whether peripheral blood responses translate to tissue-level immune activity in DM-affected organs. The microenvironmental differences between blood and lesional skin or muscle remain a key limitation in interpreting these results. Patient heterogeneity in treatment history and autoantibody presence may have influenced responses. Cross-talk between the downstream COX2 and 15LOX1 pathways was not studied. Moreover, the interactions between COX2/15LOX1 and their prostaglandin/lipoxin products with PPARγ should be more extensively studied to better understand the cross-talk between the CB2R and PPARγ pathways. Downstream activation and analysis of PPARγ was not evaluated in this study but should be investigated to better characterize the lenabasum-specific PPARγ mechanism. Due to panel restrictions, live-dead stain was not performed, as we prioritized markers relevant to the immune populations and cytokines under investigation. To mitigate this, cell viability was inferred using FSC/SSC gating to exclude dead cells and debris based on size and granularity, a standard but less specific method compared to live-dead staining. This approach was intended to minimize signals from non-viable cells, though it may not fully replicate the precision of live-dead staining and has the potential to introduce aberrant signal. This study was also conducted in DM peripheral blood alone and the contribution of fibroblast, skin-resident immune cells, or other tissue-specific factors on immune cell activation was not explored^38^. It is important to note that all the cell populations investigated have shown increased CB2R and PPARγ levels in skin compared to peripheral blood, suggesting that these effects may be more pronounced in DM skin^12^.

In this study, we map the cellular interferon response to preferential activation of CB2R and PPARγ with lenabasum treatment, highlighting heterogeneous cell to cell responses in DM blood. CD4^+^ T, moDCs, and iMs all activate a CB2R-mediated lenabasum pathway. This is associated with increased levels of both COX2 and 15LOX1 in CD4^+^ T cells, but only increased levels of COX2 in moDCs. B cells, CD8^+^ T, and ncMs exhibit co-dependence on both the CB2R-mediated and PPARγ-mediated lenabasum pathways. This activation of both CB2R-mediated and PPARγ-mediated lenabasum pathways is associated with increased levels of both COX2 and 15LOX1 in CD8^+^ T cells, increased levels of 15LOX1 only in B cells, and increased levels of COX2 only in ncMs. Myeloid DCs were the only investigated cells in which lenabasum independently activates the CB2R or the PPARγ pathway while also recruiting both COX2 and 15LOX1 enzymes. Myeloid DCs show the most number of possible pathways affected by lenabasum, which is useful as mDCs are known to be major drivers of DM. In addition, we demonstrate increased cellular CB2R expression in CB2R dominant cell types among lenabasum responders, suggesting heightened sensitivity and response to this treatment. Knowledge of various pathway activations across cell populations will be useful in therapeutic baseline receptor testing to guide precision medicine or for future endeavors into lenabasum’s mechanism that can potentially translate to other autoimmune skin diseases and progress the field of dermatological treatment.

## Supplementary Information

Supplementary Table S1.

Supplementary Figure S1.

Supplementary Figure S2.

Supplementary Figure S3.

Supplementary Figure S4.

Supplementary Figure S5.

## Data Availability

The patient whole blood datasets generated and analyzed during this study are available from the corresponding author on reasonable request.

## Abbreviations

DM: Dermatomyositis
CB2R: Cannabinoid type-2 receptor
PPARγ: Nuclear peroxisome proliferator-activated receptor-γ
IFNβ: Interferon-β
IFNγ: Interferon-γ
CDASI: Cutaneous Dermatomyositis Disease Area and Severity Index
COX2: Cyclooxygenase-2
15LOX1: 15-Lipoxygenase-1
AA: Arachidonic acid
15d-PGJ_2_: 15-Deoxy-Δ12,14-prostaglandin J2
LXA_4_: Lipoxin A4
NLRP3: Nod-like receptor protein 3
FPR2/ALX: Human formyl peptide receptor 2
IL-8: Interleukin-8
ACR: EULAR/American College of Rheumatology
RBC: Red blood cell
TLR4: Toll-like receptor 4
PBMC: Peripheral blood mononuclear cell
PMA: Phorbol 12-myristate 13 acetate
R848: Resiquimod
IQR: Interquartile range
mDC: Myeloid dendritic cell
ncMs: Non-classical monocyte
iM: Intermediate monocytes
moDC: Monocyte-derived dendritic cell
pDC: Plasmacytoid dendritic cell
cM: Classical monocyte
NK: Natural killer
