# Supplementary material for: Diverse Lenabasum pathway activation in dermatomyositis patients’ blood
Source: Sci Rep. 2025 May 18;15:17232. doi: 10.1038/s41598-025-92001-z (PMC12086228; doi:10.1038/s41598-025-92001-z)
Supplement: Supplementary file 1 — Supplementary Table S1. [file 41598_2025_92001_MOESM1_ESM.docx]

*Supplemental Table*

Table S1: Patient demographics, medications, disease characteristics, and ILD presence.

| **Patient** | | **Sex** | **Age** | **Current Medications** | **Antibodies** | **CDASI-A** | **DM Type** | **ILD** | **Prior Treatment** | **Disease Duration (Months)** | | **Labs** | |  |
| --- | --- | --- | --- | --- | --- | --- | --- | --- | --- | --- | --- | --- | --- | --- |
| **1** | F | 55 | HCQ, QC | - | 23 | C | - | AZA, MMF, HCQ, QC, PN | 210 | - | | |  |  |
| **2** | M | 60 | HCQ, MMF | + | 12 | A | - | HCQ, QC, MMF, | 260 | ANA 1:40,  repeat ANA 1:160 | | |  |  |
| **3** | F | 70 | HCQ, QC | + | 18 | A | - | MTX, PN, HCQ, QC | 176 | Anticardiolipin IgG antibody: 2 GPL; ANA +, speckled, 1:640 | |  | |  |
| **4** | F | 57 | MTX | - | 33 | HM | - | PN, HCQ, MTX, Lenabasum | 99 | - | | |  |  |
| **5** | F | 35 | - | - | 12 | A | - | HCQ, PN | 212 | - | | |  |  |
| **6** | F | 41 | HCQ | - | 15 | A | - | HCQ, QC | 83 | - | | |  |  |
| **7** | F | 53 | HCQ, MTX | - | 11 | C | - | MMF, BEM, PN, HCQ, MTX | 200 | - | | |  |  |
| **8** | M | 60 | - | + | 18 | C | - | PN, MTX | 32 | Positive P155/140 antibody | | |  |  |
| **9** | F | 52 | - | - | 8 | C | - | Treatment naïve | 68 | - | | |  |  |
| **10** | F | 69 | - | + | 26 | A | - | MTX | 26 | High Positive for SAE1 antibody, ANA 1:160 | | |  |  |
| **11** | F | 56 | MTX | - | 20 | A | - | MMF | 29 | - | | |  |  |
| **12** | F | 64 | mPRED | + | 13 | A | + | HCQ, mPRED | 22 | High Positive for MDA5 antibodies, ANA 1:320 (diffuse) | | |  |  |
| **13** | F | 54 | BEM | - | 18 | C | + | HCQ, BEM, MTX, MMF | 80 | - | | |  |  |
| **14** | F | 35 | PN, MTX, AZA | - | 9 | C | - | PN, MTX, AZA, HCQ | 21 | - | | |  |  |

Abbreviations: HCQ: Hydroxychloroquine; QC: Quinacrine; MMF: Mycophenolate; MTX: Methotrexate; mPRED: Methylprednisolone; BEM: Belimumab; PN: Prednisone; AZA:

Azathioprine; C: Classic; A: Amyopathic; HM: Hypo-myopathic; ILD: Interstitial lung disease; CDASI-A: Cutaneous Dermatomyositis Disease Area and Severity Index-Activity.
