# Supplementary material for: Diverse Lenabasum pathway activation in dermatomyositis patients’ blood
Source: Sci Rep. 2025 May 18;15:17232. doi: 10.1038/s41598-025-92001-z (PMC12086228; doi:10.1038/s41598-025-92001-z)
Supplement: Supplementary file 2 — Supplementary Figure S1. [file 41598_2025_92001_MOESM2_ESM.docx]

200K


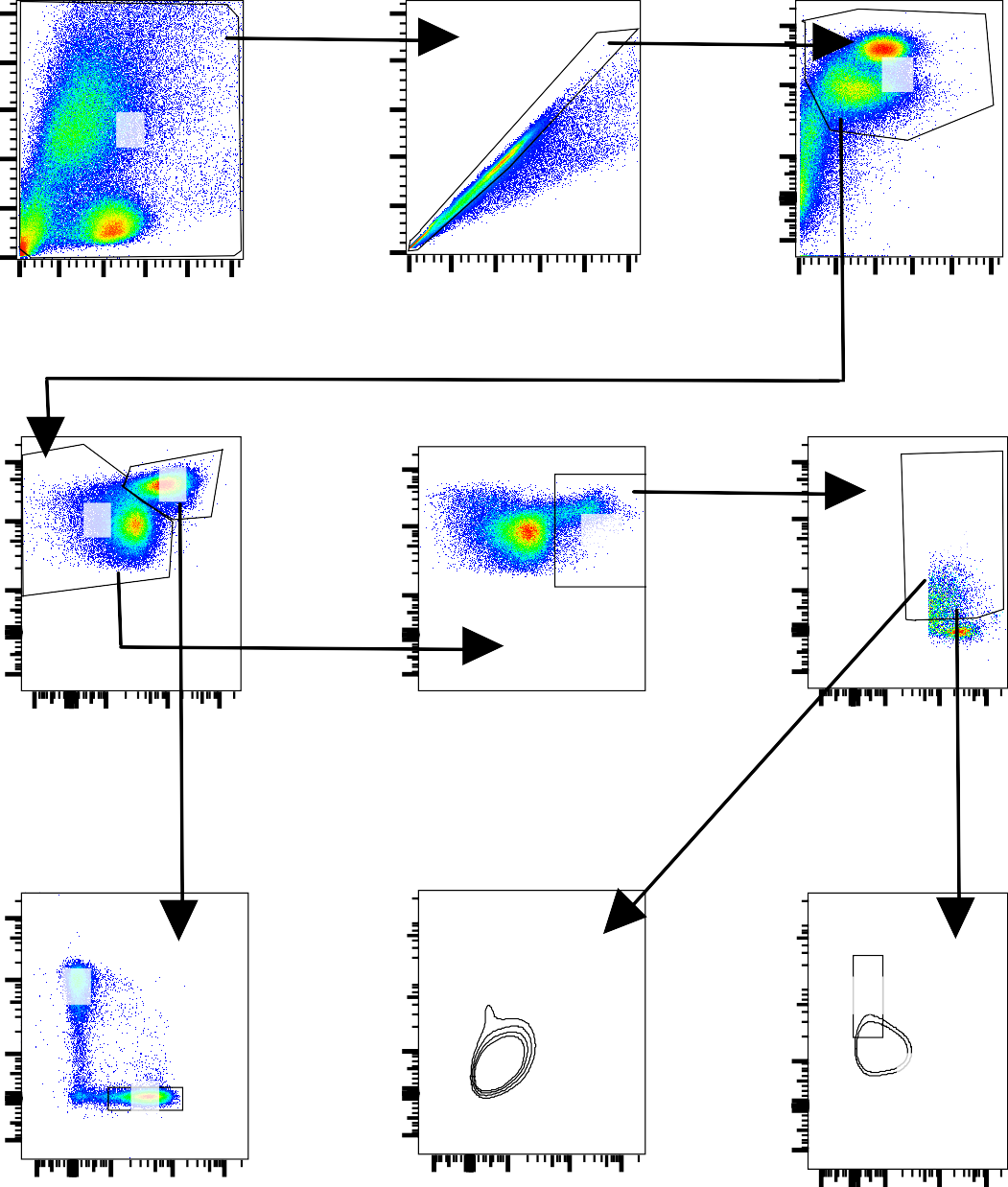

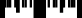

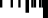

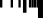

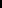


10

5

200K

10

4

CD45+

77.6

Cells

90.9

100K

0

0

0

150K

0

150K

0

150K

FSC-A

FSC-A

FSC-A

5

CD3+

44.9

10

10 5

CD3-

54.9

10

4

HLADR+

10.6

10

4

CD14+

50.1

0

0

0

10

5

0

10

5

0

10

5

CD3

HLADR

CD14

10

5

5

10

10

4

10

4

Non-Classical

11.3

CD4+

70.8

0

0

0

10

5

0

10

5

0

5

10

Classical 34.7

Intermediate 2.98

CD8+ 21.4

MoDCs+ 30.3

Single Cells 91.1

100K

FSC-H

CD45

0

10 5

4

HLADR

10

CD45

0

10 5

4

10

CD11c

CD16

0

CD4

SSC-A

CD8

CD45

**CD4+, CD8+, MoDC, nCM, iM, and cM Gating Strategy**

CD14

CD14

200K


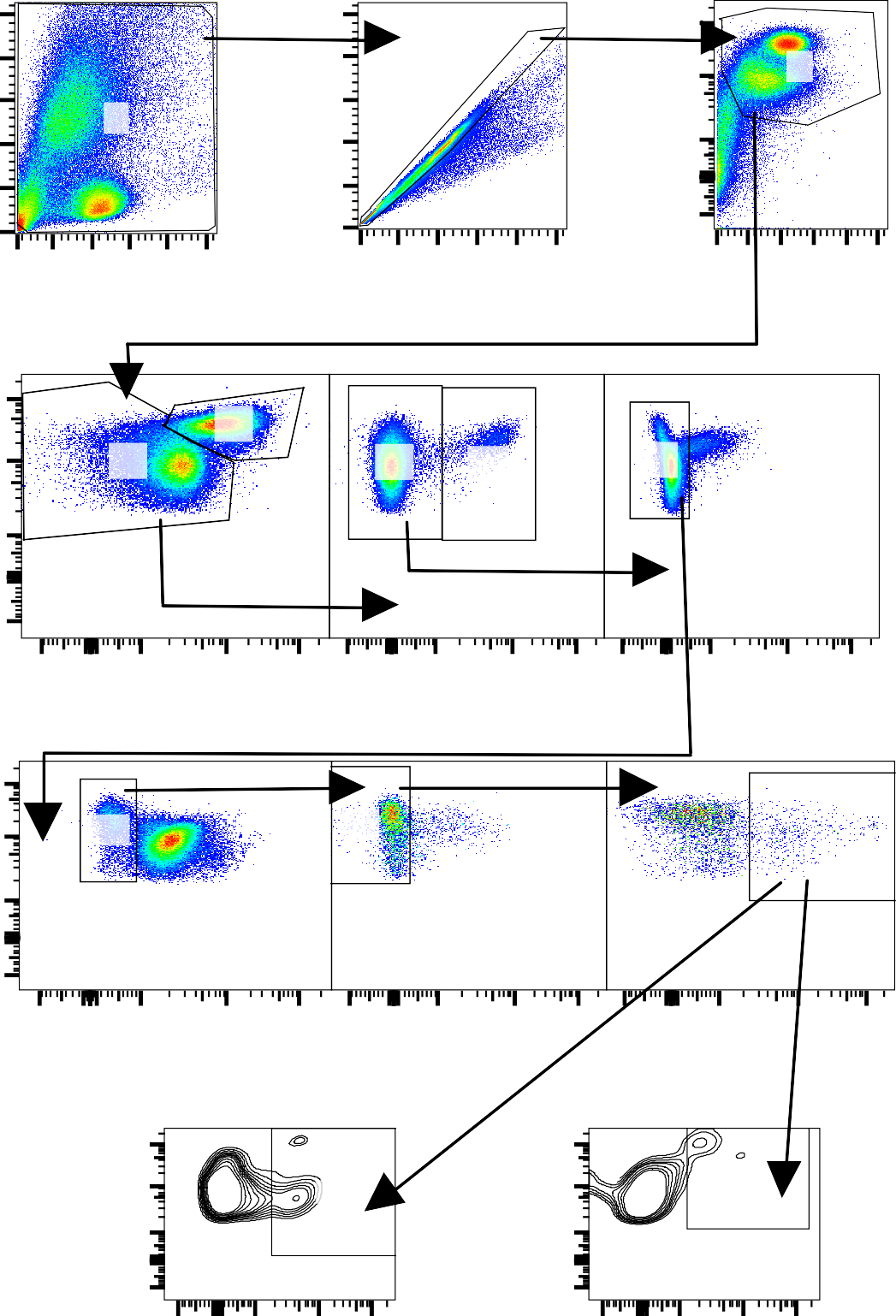


5

10

200K

4

CD45+

77.6

10

Cells

90.9

100K

0

0

0

150K

0

150K

0

150K

FSC-A

FSC-A

FSC-A

CD3+

44.9

CD3-

54.9

CD19-

96.1

CD19+

3.82

CD14-

93.2

0

10

4

0

10

4

0

10

4

CD3

CD19

CD14

CD16-

8.73

CD56-

85.3

0

10

4

0

10

4

0

10

4

CD16

CD56

HLADR

4

CD11c+

25.0

10

4

CD123+

11.5

10

0

0

HLADR+ 12.7

Single Cells 91.1

100K

SSC-A

FSC-H

CD45

0

5

10

4

10

CD45

0

5

10

4

10

CD45

0

4

CD11c

HLADR

0 10

4

0 10

HLADR CD123

**CD11c+ and CD123+ Gating Strategy**

**Figure S1:** Flow cytometry gating strategy to identify CD4^+^ T, CD8^+^ T , CD11c^+^, CD123^+^, CD19^+^, moDCs, classical monocytes, intermediate monocytes, and non-classical monocytes. A gate on total cells was set followed by a subgate to exclude doublets. CD45^+^ cells were gated on to identify leukocytes, followed by a CD3^+^ gate for CD4^+^ T and CD8^+^ T cells. CD19^+^ cells were gated through the CD3- subgate. Following the CD19- gate, the CD14- cells were gated, then the CD16- cells, then CD56- cells, and then the HLADR^+^ subgate was made to identify CD11c^+^ and CD123^+^ cells. Using the HLADR^+^ subgate, CD14^+^ cells were gated which allowed for the gating of moDCs and the monocyte subtypes: non-classical, intermediate, and classical.
