# Supplementary material for: Diverse Lenabasum pathway activation in dermatomyositis patients’ blood
Source: Sci Rep. 2025 May 18;15:17232. doi: 10.1038/s41598-025-92001-z (PMC12086228; doi:10.1038/s41598-025-92001-z)
Supplement: Supplementary file 3 — Supplementary Figure S2. [file 41598_2025_92001_MOESM3_ESM.docx]

10 5 10 5


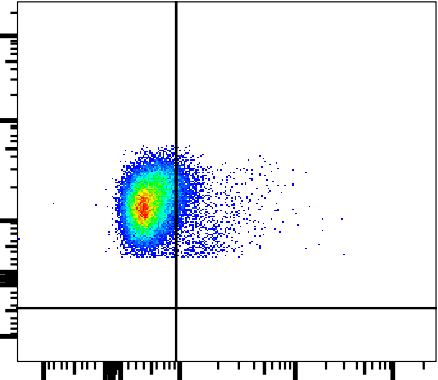


CD4+ IFNb- 92.3

Q3 0

Q4 0

Q2 7.70

Q1 92.3

CD4+ IFNb+ 7.70


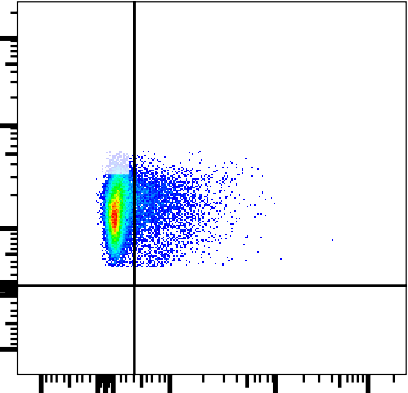


Q9 87.4

CD4+ CB2R- 87.4

Q12 0

Q11 0

Q10 12.6

CD4+ CB2R+ 12.6

10 4 10 4

CD4

CD4

10 3

0

-10 3

0 10 4

# IFNβ

10 3

0

-10 3

0 10 4

# CB2R

10 5 10 5


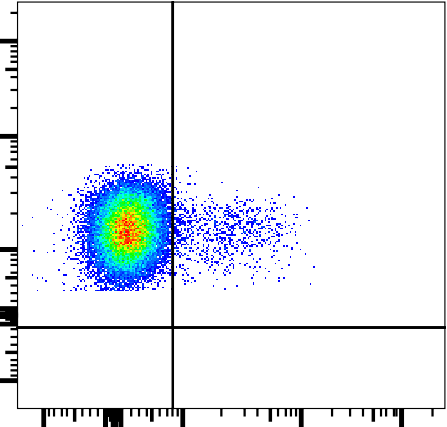


Q13 96.8

Q14 3.23

CD4+ IFNg- 96.8

Q16 0

Q15

0

CD4+ IFNg+ 3.23


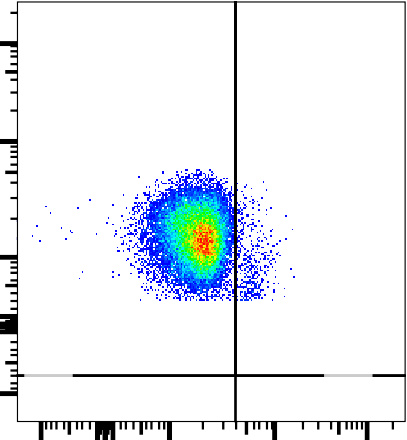


Q21 98.3

Q22 1.70

CD4+ 15LOX- 98.3

Q24 0

Q23 0

CD4+ 15LOX+ 1.70

10 4 10 4

CD4

CD4

10 3

0

-10 3

0 10 4

# IFNγ

10 3

0

-10 3

0 10 4

# 15LOX

10 5


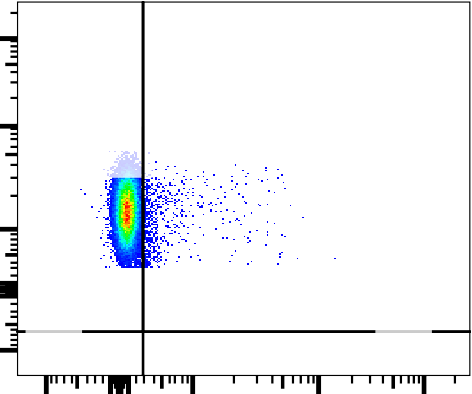


Q6 2.74

CD4+ COX2- 97.3

Q7 0

Q8 0

Q5 97.3

CD4+ COX2+ 2.74

10 4

CD4

10 3

0

-10 3

**Representative IFN, CB2R, 15LOX, and COX2 Gating Strategy**

**Figure S2**: Representative flow cytometry cytokine gating strategy using CD4^+^ T cells. The same gating strategy to determine IFNβ, ΙFNγ, CB2R, 15LOX, and COX2 levels was followed for all cell types.

0 10 4 10 5

COX2
