# Supplementary material for: Diverse Lenabasum pathway activation in dermatomyositis patients’ blood
Source: Sci Rep. 2025 May 18;15:17232. doi: 10.1038/s41598-025-92001-z (PMC12086228; doi:10.1038/s41598-025-92001-z)
Supplement: Supplementary file 4 — Supplementary Figure S3. [file 41598_2025_92001_MOESM4_ESM.docx]

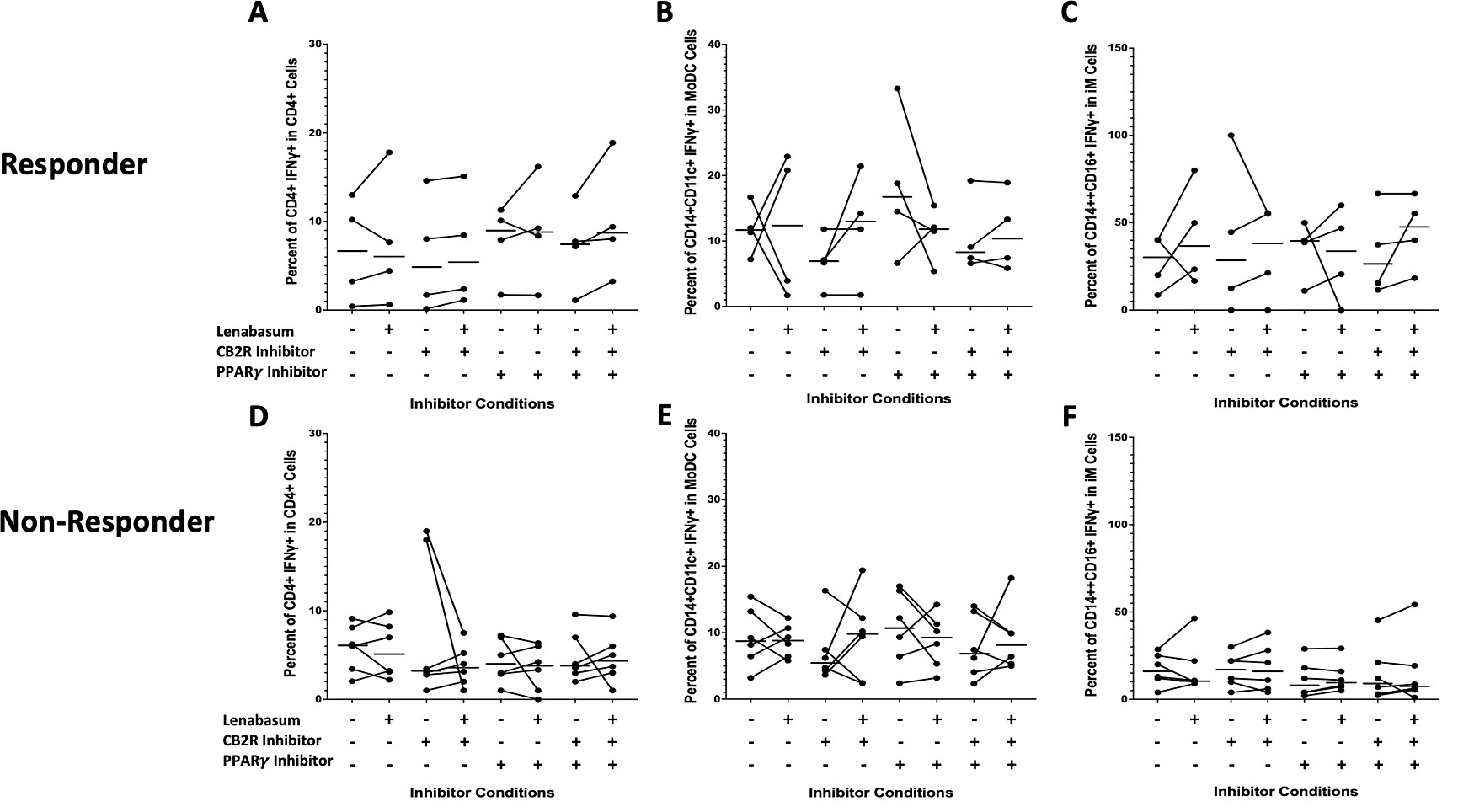


**Figure S3:** Flow cytometry of DM whole blood leukocytes displaying percent positivity of IFNγ across CD4^+^ T , moDCs, and iMs in non-responders and responders with various CB2R/PPARγ inhibition and lenabasum treatment. Black line reflects the median. A) IFNγ positivity in CD4T^+^ cells in responders with lenabasum treatment and CB2R/PPARγ inhibition. B) IFNγ positivity in moDCs in responders with lenabasum treatment and CB2R/PPARγ inhibition. C) IFNγ positivity in iMs in responders with lenabasum treatment and CB2R/PPARγ inhibition. D) IFNγ positivity in CD4T^+^ in non-responders with lenabasum treatment and CB2R/PPARγ inhibition. E) IFNγ positivity in moDCs in non-responders with lenabasum treatment and CB2R/PPARγ inhibition. F) IFNγ positivity in iMs in non-responders with lenabasum treatment and CB2R/PPARγ inhibition.
